# Dynamic decline in estimated glomerular filtration rate associated with in-hospital mortality risk in acute ischemic stroke patients after endovascular therapy: evidence from a Chinese stroke center

**DOI:** 10.3389/fnagi.2025.1598371

**Published:** 2025-11-06

**Authors:** Yanping Lin, Jingjing She, Lijuan Cai, Lingfeng Yu, Shouyue Jin, Xingyu Chen, Weiwei Gao, Renjing Zhu

**Affiliations:** 1 Department of Neurology, Zhongshan Hospital of Xiamen University, School of Medicine, Xiamen University, Xiamen, China; 2 The School of Clinical Medicine, Fujian Medical University, Fuzhou, Fujian, China; 3 Xiamen Clinical Research Center for Cerebrovascular Diseases, Xiamen, China; 4 Xiamen Quality Control Center for Stroke, Xiamen, China; 5 School of Medicine, Xiamen University, Xiamen, China

**Keywords:** acute ischemic stroke, large vessel occlusion, endovascular therapy, estimated glomerular filtration rate, dynamic change, in-hospital mortality

## Abstract

**Objectives:**

To investigate the association between dynamic changes in estimated glomerular filtration rate (eGFR) and in-hospital mortality risk in patients with acute ischemic stroke due to large vessel occlusion (LVO-AIS) undergoing endovascular therapy (EVT).

**Methods:**

This retrospective cohort study consecutively enrolled 329 patients with anterior circulation LVO-AIS who underwent EVT between January 2018 and January 2025. The eGFR was calculated using the Chronic Kidney Disease Epidemiology Collaboration (CKD-EPI) 2009 equation at admission (baseline), and on days 1 and 3 post-EVT. The primary outcome was all-cause in-hospital mortality. Multivariable logistic regression models and restricted cubic spline regression were employed to assess the association between eGFR and outcomes. Subgroup analyses with interaction testing were conducted to evaluate the consistency of this association across different patient populations.

**Results:**

Of the 329 patients, 49 (14.9%) died during hospitalization. Baseline eGFR was not significantly associated with mortality (*P* = 0.223), whereas post-EVT eGFR demonstrated a pronounced time-dependent association. Patients who died exhibited a progressive decline in eGFR (*P* < 0.05), while survivors showed a modest increase (*P* < 0.01). After comprehensive adjustment for confounders, each 1 mL/min/1.73 m^2^ decrease in day-3 eGFR was associated with a 3% increase in mortality risk (*P* < 0.001); moderate-to-severe renal dysfunction (eGFR < 60 mL/min/1.73 m^2^) on day 3 was associated with a 4.3-fold increased risk of death (*P* < 0.001). Subgroup analyses revealed consistent associations across subgroups, with no significant interactions (all P for interaction > 0.05). Furthermore, post-EVT eGFR decline was significantly associated with increased risk of symptomatic intracerebral hemorrhage (sICH) (*P* < 0.001), but not with hemorrhagic transformation (HT).

**Conclusion:**

Dynamic decline in eGFR, particularly the level on day 3 post-EVT, is independently associated with in-hospital mortality in LVO-AIS patients undergoing EVT, exhibiting a clear dose-response relationship.

## 1 Introduction

Acute ischemic stroke (AIS) remains a leading cause of death and disability worldwide, with large vessel occlusion (LVO) accounting for approximately 28%–46% of all AIS cases (Feigin et al., 2025; Smith et al., 2009). Patients with LVO-AIS represent a critical population for clinical intervention due to their higher mortality, greater disability burden, and poorer functional outcomes (Malhotra et al., 2017). Multiple landmark randomized controlled trials have demonstrated that endovascular therapy (EVT) significantly improves clinical outcomes in LVO patients, establishing it as the standard of care for this population (Compagne et al., 2022; Jansen et al., 2018; Mueller-Kronast et al., 2017). Despite continuous advances in recanalization techniques, however, in-hospital mortality rates among EVT patients remain at 9%–20%, underscoring the clinical importance of identifying reliable prognostic predictors (Sluis et al., 2023; Wang et al., 2024).

A complex bidirectional pathophysiological relationship exists between renal dysfunction and AIS. Previous studies have shown that approximately 12% of stroke patients develop acute kidney injury (AKI), characterized by progressive elevation in serum creatinine, during the acute phase (Zorrilla-Vaca et al., 2018). This post-stroke AKI can be mediated through multiple mechanisms, including hemodynamic instability, hypovolemia, contrast-induced injury, sympathetic nervous system overactivation, and systemic inflammatory responses (Pesonen et al., 2021; Saeed et al., 2014). Conversely, renal dysfunction may exacerbate neurological injury through various kidney-brain axis pathways, including accumulation of neurotoxins, enhanced neuroinflammation, increased blood-brain barrier permeability, and elevated oxidative stress. These pathological changes promote the transformation of ischemic penumbra into infarct core and contribute to a cascade of complications including cerebral edema, hemorrhagic transformation, inflammatory cascades, and ischemia-reperfusion injury (Chen et al., 2024). As a core indicator of renal function, estimated glomerular filtration rate (eGFR) has been extensively validated for its clinical value. Current evidence indicates that eGFR is not only a risk factor for cerebrovascular events in healthy populations (Oh et al., 2021), but also significantly associated with various adverse outcomes in AIS patients (Kim et al., 2015; Lee et al., 2013; Liu et al., 2020; Shi et al., 2019).

Building on this background, we measured eGFR at admission and on days 1 and 3 post-EVT, and further investigated the association between dynamic eGFR changes and in-hospital mortality risk in patients with anterior circulation LVO-AIS. We hypothesized that dynamic eGFR decline, rather than single time-point measurements, might provide more valuable information for early risk assessment in EVT patients. These findings may help optimize perioperative management strategies for patients undergoing EVT.

## 2 Materials and methods

### 2.1 Study population

This was a retrospective cohort study based on a single-center stroke registry. We consecutively enrolled adult patients with anterior circulation LVO-AIS who underwent EVT at our center between January 2018 and January 2025. Anterior circulation LVO was defined as occlusion of the internal carotid artery terminus or middle cerebral artery M1/M2 segments, with all diagnoses confirmed by computed tomography angiography, magnetic resonance angiography, or intraoperative digital subtraction angiography (DSA). Exclusion criteria included: (1) intracranial hemorrhage on baseline neuroimaging; (2) pre-stroke modified Rankin Scale (mRS) score > 2; and (3) missing key clinical data required for analysis. The study protocol was approved by the institutional ethics committee and conducted in accordance with the Declaration of Helsinki. Given the retrospective nature of the study and the use of de-identified data, the ethics committee waived the requirement for patient informed consent.

### 2.2 Data collection

We extracted baseline clinical data from the stroke registry, including demographic characteristics (age and sex), cerebrovascular risk factors (smoking status, alcohol consumption, hypertension, diabetes mellitus, hyperlipidemia, atrial fibrillation, previous stroke or transient ischemic attack, and coronary heart disease), admission blood pressure, and baseline neurological assessment. Neurological status was evaluated using the National Institutes of Health Stroke Scale (NIHSS). The extent of early ischemic changes was assessed using the Alberta Stroke Program Early CT Score (ASPECTS). Stroke etiology was classified according to the Trial of Org 10172 in Acute Stroke Treatment (TOAST) criteria.

We documented detailed procedural information, including pre-EVT intravenous thrombolysis, key time metrics (onset-to-puncture time, onset-to-reperfusion time, and puncture-to-reperfusion time), and technical details of the intervention. Technical parameters included the number of mechanical thrombectomy passes, device strategy (stent retriever, aspiration catheter, or combined approach), use of rescue treatments such as balloon angioplasty, and final reperfusion status. Reperfusion results were evaluated using the modified Thrombolysis in Cerebral Ischemia (mTICI) scoring system based on final DSA findings, with mTICI grades 2b-3 defined as successful reperfusion.

Laboratory indicators were collected at three time points during hospitalization: at admission (baseline, Day 0), and on the mornings of day 1 and day 3 post-EVT after overnight fasting. Laboratory tests included complete blood counts (white blood cells, neutrophils, lymphocytes, monocytes, red blood cells, hemoglobin, and platelets), biochemical parameters (total protein, albumin, aspartate aminotransferase, alanine aminotransferase, creatinine, and uric acid), and lipid profiles (total cholesterol, triglycerides, high-density lipoprotein cholesterol, and low-density lipoprotein cholesterol). All laboratory tests were performed in the hospital’s central laboratory according to standardized operating procedures, with regular calibration and quality control.

### 2.3 Exposure factors

We calculated the estimated glomerular filtration rate (eGFR) using the Chronic Kidney Disease Epidemiology Collaboration (CKD-EPI) 2009 equation:


$$e\,G\,F\,R\,\left(m\,l/m\,i\,n/1.73\,m^{\text{2}}\right)=a\times S\,c\,r/b^{c}\times 0.993^{a\,g\,e}$$

where for females: *a* = 144, *b* = 0.7, *c* = −0.329 when Scr ≤ 0.7 mg/dL, and *c* = −1.209 when Scr > 0.7 mg/dL; for males: *a* = 141, *b* = 0.9, *c* = −0.411 when Scr ≤ 0.9 mg/dL, and *c* = −1.209 when Scr > 0.9 mg/dL. Serum creatinine conversion: 1 mg/dL = 88.49 μmol/L.

We selected the CKD-EPI formula rather than the Modification of Diet in Renal Disease equation because the former has demonstrated superior accuracy and less bias across various populations, particularly in the eGFR > 60 mL/min/1.73 m^2^ range, and is widely applied in patients with acute cerebrovascular disease. Following the recommendations of the 2024 Kidney Disease: Improving Global Outcomes (KDIGO) Clinical Practice Guideline for Chronic Kidney Disease (Kidney Disease: Improving Global Outcomes (KDIGO) CKD Work Group, 2024), we dichotomized eGFR levels into normal (≥ 60 mL/min/1.73 m^2^) and moderate-to-severe decline (< 60 mL/min/1.73 m^2^). To explore the dose-response relationship between eGFR and outcomes, we stratified patients into quartiles (Q1–Q4) based on the eGFR distribution at each time point in the study population.

### 2.4 Outcome assessment

The primary outcome of this study was in-hospital all-cause mortality, defined as death from any cause during the index hospitalization. All mortality cases were independently reviewed by two neurological specialists to confirm the cause of death. Secondary outcomes included hemorrhagic transformation (HT) and symptomatic intracerebral hemorrhage (sICH). All patients routinely underwent cranial CT examinations immediately after EVT, and at 24 and 72 h post-procedure. Additional CT scans were performed immediately when patients exhibited neurological deterioration or when previous imaging suggested progression of edema.

Hemorrhagic transformation was classified according to the European Cooperative Acute Stroke Study criteria as: (1) HI1 (small petechial hemorrhage); (2) HI2 (confluent petechiae without mass effect); (3) PH1 (parenchymal hematoma ≤ 30% of the infarcted area with mild mass effect); and (4) PH2 (parenchymal hematoma > 30% of the infarcted area with significant mass effect). sICH was defined as any intracranial hemorrhage accompanied by one of the following conditions without other explicable causes: (1) an increase in total NIHSS score ≥ 4 points from baseline; (2) an increase in any single NIHSS item ≥ 2 points from baseline; or (3) neurological deterioration requiring endotracheal intubation, decompressive craniectomy, external ventricular drainage, or other significant medical interventions. All imaging assessments were independently performed by two experienced neuroradiologists who were blinded to clinical data, with disagreements resolved by a third expert.

### 2.5 Statistical analysis

All statistical analyses were performed using R software (version 4.2.2). The normality of continuous variables was assessed using the Shapiro-Wilk test and quantile-quantile plots. Normally distributed continuous variables were presented as mean ± standard deviation (mean ± SD) and compared using the independent samples *t*-test, while non-normally distributed continuous variables were presented as median [interquartile range (IQR)] and compared using the Mann-Whitney *U*-test. Categorical variables were expressed as frequencies (percentages) and compared using Pearson’s chi-squared test or Fisher’s exact test (when expected cell counts were < 5).

To evaluate the independent association between eGFR and in-hospital mortality, we constructed three progressively adjusted multivariable logistic regression models. Covariates were selected based on the results of univariate analysis (*P* < 0.05). We assessed multicollinearity among variables using variance inflation factor (VIF) and tolerance values, with VIF < 10 and tolerance > 0.1 considered acceptable ranges (Supplementary Table 1). Model 1 was the unadjusted baseline model; Model 2 adjusted for demographic and clinical factors (age, smoking status, atrial fibrillation, and coronary heart disease); Model 3 further adjusted for procedural parameters and laboratory indicators (baseline NIHSS score, puncture-to-reperfusion time, lymphocyte count, platelet count, and aspartate aminotransferase). Results were presented as odds ratios (OR) with 95% confidence intervals (95% CI).

To explore potential non-linear associations between eGFR and in-hospital mortality, we employed restricted cubic spline (RCS) regression models with knots placed at the 5th, 35th, 65th, and 95th percentiles of eGFR. Based on previous research evidence and biological plausibility, we conducted subgroup analyses according to sex, age (< 60 vs. ≥ 60 years), and vascular risk factors, and assessed between-subgroup effect heterogeneity using interaction tests. All statistical tests were two-sided, with *P* < 0.05 considered statistically significant.

## 3 Results

### 3.1 Patient characteristics and laboratory parameters

The study included 329 patients with anterior circulation LVO-AIS who underwent EVT, of whom 49 (14.9%) died during hospitalization. Table 1 presents a comparison of baseline characteristics between groups. Patients in the mortality group were significantly older (*P* = 0.002), had higher baseline NIHSS scores (*P* = 0.009), and exhibited higher proportions of atrial fibrillation (*P* = 0.004) and coronary heart disease (*P* = 0.035), but lower rates of current smoking (*P* = 0.019). Regarding procedural characteristics, the mortality group demonstrated significantly prolonged puncture-to-reperfusion time (*P* = 0.002).

**TABLE 1:** Baseline characteristics of patients stratified by in-hospital mortality after endovascular therapy for acute ischemic stroke.

Variables	Overall (*n* = 329)	Survival group (*n* = 280)	Mortality group (*n* = 49)	*P*-value
Age, years	68 (58, 77)	68 (57, 76)	72 (59, 82)	0.002
Male sex	216 (65.7)	187 (66.8)	29 (59.2)	0.301
Current smoking status	123 (37.4)	112 (40.0)	11 (22.4)	0.019
Alcohol consumption	83 (25.2)	70 (25.0)	13 (26.5)	0.820
**Medical history**				
Hypertension	222 (67.5)	186 (66.4)	36 (73.5)	0.332
Diabetes mellitus	104 (31.6)	87 (31.1)	17 (34.7)	0.615
Hyperlipidemia	74 (22.5)	63 (22.5)	11 (22.4)	0.994
Atrial fibrillation	146 (44.4)	115 (41.1)	31 (63.3)	0.004
Previous stroke or TIA	54 (16.4)	48 (17.1)	6 (12.2)	0.393
Coronary artery disease	43 (13.1)	32 (11.4)	11 (22.4)	0.035
**Clinical presentation**				
Admission SBP, mmHg	149 (132, 163)	149 (132, 162)	150 (138, 167)	0.349
Admission DBP, mmHg	87 (76, 96)	86 (76, 97)	87 (79, 92)	0.663
Baseline NIHSS score	15 (12, 19)	14 (11, 18)	17 (14, 20)	0.009
Baseline ASPECT score	9 (8, 10)	9 (8, 10)	9.00 (7, 10)	0.113
**Etiology of stroke**				0.073
Large-artery atherosclerosis	161 (48.9)	143 (51.1)	18 (36.7)	–
Cardioembolism	152 (46.2)	122 (43.6)	30 (61.2)	–
Other	16 (4.9)	15 (5.4)	1 (2.0)	–
**Procedural characteristics**				
Intravenous thrombolysis	136 (41.3)	116 (41.4)	20 (40.8)	0.936
OPT, min	365 (262, 559)	367 (259, 560)	362 (287, 496)	0.549
PRT, min	78 (50, 100)	75 (45, 98)	86 (61, 126)	0.002
ORT, min	451 (335, 638)	453 (327, 642)	435 (358, 629)	0.670
NOTA	2.00 (1.00, 2.00)	2.00 (1.00, 2.00)	2.00 (1.00, 3.00)	0.220
Successful reperfusion	275 (83.6)	236 (84.3)	39 (79.6)	0.413
**Treatment strategy**				0.608
Stent retriever	68 (20.7)	57 (20.4)	11 (22.4)	–
Aspiration	21 (6.4)	16 (5.7)	5 (10.2)	–
Combined approach	218 (66.3)	188 (67.1)	30 (61.2)	–
Balloon angioplasty	62 (18.8)	51 (18.2)	11 (22.4)	0.484

Values are presented as median (interquartile range) or number (percentage). TIA, transient ischemic attack; SBP, systolic blood pressure; DBP, diastolic blood pressure; NIHSS, National Institutes of Health Stroke Scale; ASPECTS, Alberta Stroke Program Early CT Score; OPT, onset-to-puncture time; PRT, puncture-to-reperfusion time; ORT, onset-to-reperfusion time; NOTA, number of thrombectomy attempts. Combined approach refers to the use of both stent retriever and aspiration techniques during the procedure.

**FIGURE 1:**
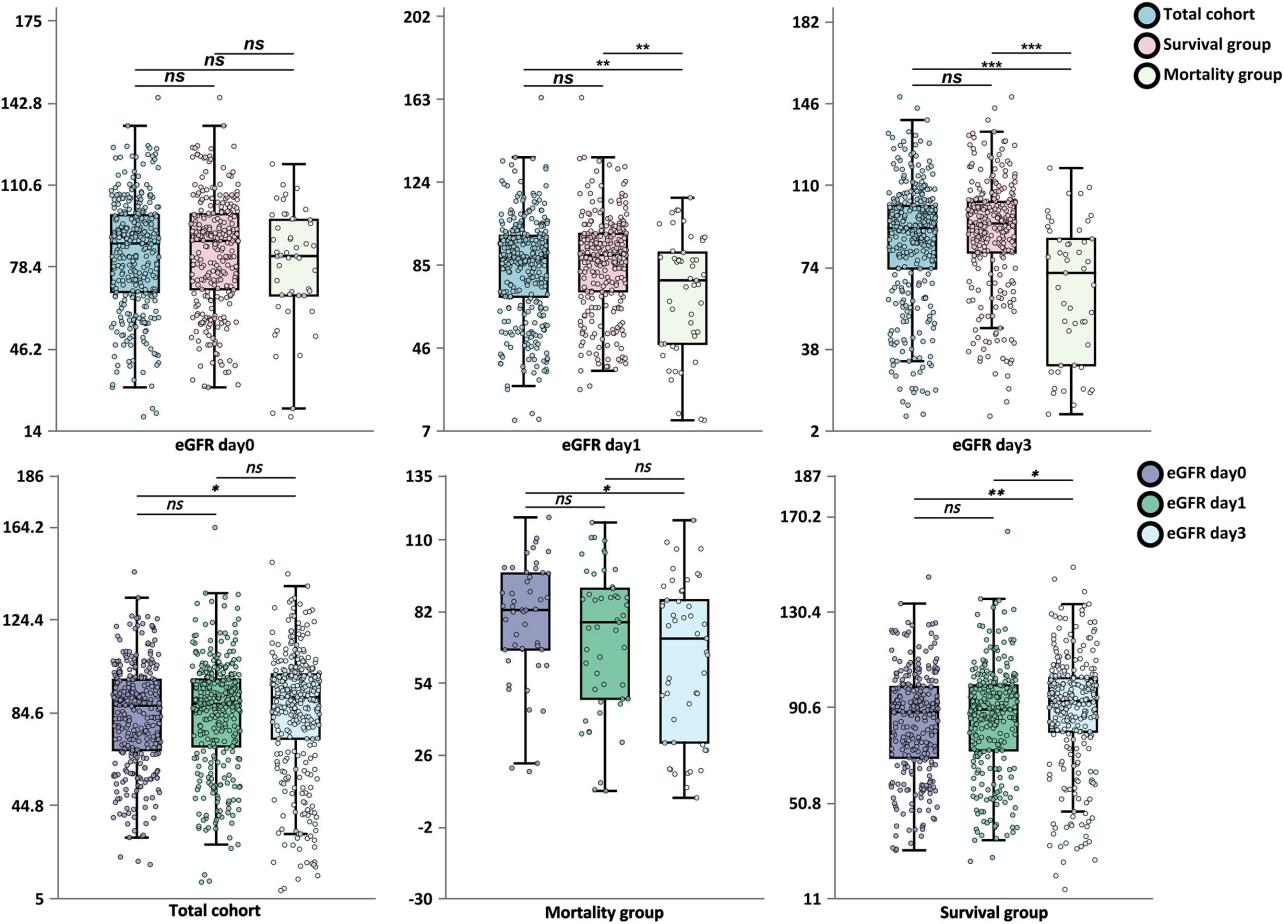
Temporal changes in estimated glomerular filtration rate after endovascular therapy according to clinical outcome. Box-and-whisker plots depict the distribution of eGFR at admission (day 0) and on days 1 and 3 post-EVT, stratified by survival status. Data are shown for the total cohort (*n* = 329), survivors (*n* = 280), and non-survivors (*n* = 49). Each dot represents an individual patient. Within each box, the horizontal line represents the median; box boundaries represent the interquartile range (IQR; 25th to 75th percentiles); and whiskers extend to the most extreme values within 1.5 × IQR. Between-group comparisons were performed using the Mann-Whitney *U*-test; within-group longitudinal comparisons used the Wilcoxon signed-rank test. ns, not significant; *P < 0.05; **P < 0.01; ***P < 0.001. eGFR, estimated glomerular filtration rate; EVT, endovascular therapy.

Table 2 illustrates the comparison of laboratory parameters between outcome groups. Among baseline blood cell count indicators, patients in the mortality group had significantly lower lymphocyte counts compared to the survival group (*P* < 0.001) and markedly reduced platelet counts (*P* = 0.022). Regarding liver function, the mortality group exhibited lower aspartate aminotransferase levels (*P* = 0.019). Renal function indicators revealed a notable time-dependent trend. At admission (baseline), creatinine levels were comparable between both groups, with no statistical difference (*P* = 0.687). However, by day 1 post-EVT, creatinine levels began to rise in the mortality group (*P* = 0.030), with the difference further amplified by day 3 (*P* < 0.001) (Figure 1).

**TABLE 2:** Comparison of laboratory parameters among patients stratified by in-hospital mortality after endovascular therapy for acute ischemic stroke.

Variables	Overall (*n* = 329)	Survival group (*n* = 280)	Mortality group (*n* = 49)	*P*-value
White blood cell, × 10^9^/L	8.2 (6.5, 10.3)	8.2 (6.6, 10.0)	9.0 (6.4, 12.7)	0.205
Neutrophils, × 10^9^/L	5.71 (4.22, 7.90)	5.66 (4.21, 7.56)	6.10 (4.42, 10.36)	0.114
Lymphocyte, × 10^9^/L	1.52 (1.14, 2.13)	1.60 (1.18, 2.19)	1.24 (0.85, 1.50)	<0.001
Monocytes, × 10^9^/L	0.45 (0.34, 0.56)	0.45 (0.34, 0.56)	0.45 (0.29, 0.60)	0.652
Red blood cell, × 10^12^/L	4.43 (4.09, 4.89)	4.45 (4.10, 4.92)	4.42 (3.86, 4.74)	0.192
Hemoglobin, g/L	137 (124, 149)	137 (124, 149)	138 (116, 148)	0.211
Platelet count, × 10^9^/L	202 (166, 237)	205 (170, 238)	176 (143, 219)	0.022
Total protein, g/L	71 (68, 75)	71 (68, 74)	72 (67, 78)	0.207
Albumin, g/L	39.1 (37.3, 41.9)	39.0 (37.3, 41.5)	40.3 (37.4, 42.4)	0.252
AST, U/L	15 (11, 23)	16 (11, 23)	13 (9, 19)	0.019
ALT, U/L	24 (19, 30)	24 (19, 30)	22 (19, 27)	0.313
Triglycerides, mmol/L	1.13 (0.86, 1.76)	1.13 (0.86, 1.71)	1.21 (0.85, 1.95)	0.536
Total cholesterol, mmol/L	4.75 (3.82, 5.32)	4.75 (3.90, 5.36)	4.47 (3.78, 5.20)	0.420
HDL cholesterol, mmol/L	1.16 (1.00, 1.36)	1.18 (1.02, 1.36)	1.09 (0.92, 1.36)	0.098
LDL cholesterol, mmol/L	3.03 (2.47, 3.61)	3.03 (2.48, 3.63)	3.07 (2.35, 3.59)	0.552
Uric acid, μmol/L	6.30 (4.87, 7.90)	6.23 (4.80, 7.40)	6.81 (5.20, 8.45)	0.252
**Creatinine, μmol/L**				
Day 0	74 (61, 89)	73 (61, 89)	74 (62, 88)	0.687
Day 1	72 (59, 86)	72 (59, 85)	79 (58, 126)	0.030
Day 3	68 (54, 85)	65 (53, 81)	93 (67, 143)	<0.001

Data are presented as median (interquartile range). Day 0 indicates values at admission (baseline); Day 1, values on the first day after endovascular therapy; and Day 3, values on the third day after endovascular therapy. AST indicates aspartate aminotransferase; ALT, alanine aminotransferase; HDL, high-density lipoprotein; LDL, low-density lipoprotein.

### 3.2 Relationship between eGFR and in-hospital mortality

Table 3 demonstrates the association between eGFR levels at different time points and in-hospital mortality. At admission, there was no significant difference in median eGFR between the mortality and survival groups (83 vs. 89 mL/min/1.73 m^2^, *P* = 0.170). However, by day 1 post-EVT, eGFR levels in the mortality group began to significantly decline (78 vs. 90 mL/min/1.73 m^2^, *P* = 0.001). By day 3 post-procedure, the eGFR difference between groups further widened (71 vs. 93 mL/min/1.73 m^2^, *P* < 0.001).

**TABLE 3:** Temporal changes in eGFR and their association with in-hospital mortality in patients with acute ischemic stroke after endovascular therapy.

Variables	Overall (*n* = 329)	Survival group (*n* = 280)	Mortality group (*n* = 49)	*P*-value
eGFR day 0	88 (68, 99)	89 (70, 99)	83 (67, 97)	0.170
eGFR day 1	88 (70, 99)	90 (73, 100)	78 (48, 91)	0.001
eGFR day 3	91 (73, 101)	93 (80, 103)	71 (31, 86)	<0.001
eGFR day 0				0.751
≥60	270 (82.07)	229 (81.79)	41 (83.67)	–
<60	59 (17.93)	51 (18.21)	8 (16.33)	–
**eGFR day 1**				0.002
≥60	268 (81.46)	236 (84.29)	32 (65.31)	–
<60	61 (18.54)	44 (15.71)	17 (34.69)	–
**eGFR day 3**				<0.001
≥60	268 (81.46)	241 (86.07)	27 (55.10)	–
<60	61 (18.54)	39 (13.93)	22 (44.90)	–
**eGFR day 0**				<0.001
Q1	144 (43.77)	129 (46.07)	15 (30.61)	–
Q2	126 (38.30)	100 (35.71)	26 (53.06)	–
Q3	40 (12.16)	37 (13.21)	3 (6.12)	–
Q4	16 (4.86)	14 (5.00)	2 (4.08)	–
**eGFR day 1**				0.002
Q1	150 (45.59)	137 (48.93)	13 (26.53)	–
Q2	118 (35.87)	99 (35.36)	19 (38.78)	–
Q3	21 (6.38)	15 (5.36)	6 (12.24)	–
Q4	5 (1.52)	2 (0.71)	3 (6.12)	–
**eGFR day 3**				<0.001
Q1	170 (51.67)	159 (56.79)	11 (22.45)	–
Q2	98 (29.79)	82 (29.29)	16 (32.65)	–
Q3	24 (7.29)	16 (5.71)	8 (16.33)	–
Q4	16 (4.86)	5 (1.79)	11 (22.45)	–

Data are presented as median (interquartile range) for continuous variables and number (percentage) for categorical variables. For quartile analyses, patients were categorized based on eGFR distribution at each time point: at admission (day 0), Q1, 19.5 to < 68.5; Q2, 68.5 to < 87.5; Q3, 87.5 to < 98.6; Q4, 98.6 to 145 mL/min/1.73 m^2^; at day 1 post-EVT, Q1, 12.0 to < 70.1; Q2, 70.1 to < 88.3; Q3, 88.3 to < 98.8; Q4, 98.8 to 164 mL/min/1.73 m^2^; at day 3 post-EVT, Q1, 8.4 to < 73.4; Q2, 73.4 to < 91.0; Q3, 91.0 to < 101; Q4, 101 to 149 mL/min/1.73 m^2^. Q1 represents the lowest eGFR values and Q4 the highest. eGFR, estimated glomerular filtration rate; EVT, endovascular therapy.

When stratifying eGFR levels using 60 mL/min/1.73 m^2^ as the threshold, the proportion of patients with moderate-to-severe renal impairment (< 60 mL/min/1.73 m^2^) at admission was similar between groups (mortality group: 16.33% vs. survival group: 18.21%, *P* = 0.751). However, following EVT, the proportion of patients with moderate-to-severe renal impairment in the mortality group increased significantly, reaching 34.69% on day 1 (vs. survival group: 15.71%, *P* = 0.002) and further rising to 44.90% by day 3 (vs. survival group: 13.93%, *P* < 0.001).

To further investigate the relationship between eGFR and in-hospital mortality, we analyzed patients according to eGFR quartiles. At admission, the distribution of eGFR quartiles did not differ significantly between the mortality and survival groups (*P* = 0.225). By day 1 post-EVT, the eGFR quartile distribution became significantly different between groups (*P* = 0.004), with Q1 markedly overrepresented in the mortality group (42.9%), while higher quartiles (Q3 and Q4) were underrepresented (14.3% each). This trend became even more pronounced by day 3 post-EVT (*P* < 0.001), with Q1 accounting for 53.1% of the mortality group, while Q4 represented only 8.2%.

Figure 1 further illustrates the dynamic patterns of eGFR change. Patients in the mortality group exhibited a marked time-dependent declining trend (baseline vs. day 3, *P* < 0.05), with progressively decreasing eGFR; conversely, the survival group demonstrated an upward trend (day 0 vs. day 3, *P* < 0.01).

### 3.3 Association between eGFR and hemorrhagic complications

Table 4 illustrates the association between eGFR levels at different time points and hemorrhagic complications (HT and sICH). At baseline, eGFR levels showed no significant association with either HT or sICH, whether analyzed as a continuous variable (HT group vs. non-HT group: *P* = 0.316; sICH group vs. non-sICH group: *P* = 0.789), as a dichotomized variable (HT: *P* = 0.338; sICH: *P* = 0.472), or by quartiles (HT: *P* = 0.629; sICH: *P* = 0.531).

**TABLE 4:** Association between dynamic changes in eGFR and hemorrhagic complications in patients with acute ischemic stroke after endovascular therapy.

Variables	HT		*P-*value	sICH		*P-*value
	No, *N* = 171	Yes, *N* = 158		No, *N* = 262	Yes, *N* = 67	
eGFR day 0	86 (67, 97)	89 (69, 99)	0.316	87 (69, 97)	89 (67, 99)	0.789
eGFR day 1	87 (72, 98)	90 (68, 101)	0.458	88 (72, 98)	87 (53, 103)	0.619
eGFR day 3	92 (79, 101)	90 (67, 102)	0.543	92 (79, 101)	88 (50, 101)	0.082
**eGFR day 0**	–	–	0.338	–	–	0.472
≥60	137 (80.12)	133 (84.18)	–	213 (81.30)	57 (85.07)	–
<60	34 (19.88)	25 (15.82)	–	49 (18.70)	10 (14.93)	–
**eGFR day 1**	–	–	0.105	–	–	0.021
≥60	145 (84.80)	123 (77.85)	–	220 (83.97)	48 (71.64)	–
<60	26 (15.20)	35 (22.15)	–	42 (16.03)	19 (28.36)	–
**eGFR day 3**	–	–	0.105	–	–	<0.001
≥60	145 (84.80)	123 (77.85)	–	223 (85.11)	45 (67.16)	–
<60	26 (15.20)	35 (22.15)	–	39 (14.89)	22 (32.84)	–
**eGFR day 0**	–	–	0.629	–	–	0.531
Q1	44 (25.73)	38 (24.05)	–	63 (24.05)	19 (28.36)	–
Q2	45 (26.32)	37 (23.42)	–	68 (25.95)	14 (20.90)	–
Q3	44 (25.73)	38 (24.05)	–	68 (25.95)	14 (20.90)	–
Q4	38 (22.22)	45 (28.48)	–	63 (24.05)	20 (29.85)	–
**eGFR day 1**	–	–	0.197	–	–	0.112
Q1	41 (23.98)	41 (25.95)	–	59 (22.52)	23 (34.33)	–
Q2	51 (29.82)	31 (19.62)	–	70 (26.72)	12 (17.91)	–
Q3	39 (22.81)	43 (27.22)	–	69 (26.34)	13 (19.40)	–
Q4	40 (23.39)	43 (27.22)	–	64 (24.43)	19 (28.36)	–
**eGFR day 3**	–	–	0.480	–	–	0.060
Q1	38 (22.22)	44 (27.85)	–	58 (22.14)	24 (35.82)	–
Q2	42 (24.56)	40 (25.32)	–	66 (25.19)	16 (23.88)	–
Q3	48 (28.07)	34 (21.52)	–	72 (27.48)	10 (14.93)	–
Q4	43 (25.15)	40 (25.32)	–	66 (25.19)	17 (25.37)	–

Data are presented as median (interquartile range) for continuous variables and number (percentage) for categorical variables. For quartile analyses, patients were categorized based on eGFR distribution at each time point: at admission (day 0), Q1, 19.5 to < 68.5; Q2, 68.5 to < 87.5; Q3, 87.5 to < 98.6; Q4, 98.6 to 145 mL/min/1.73 m^2^; at day 1 post-EVT, Q1, 12.0 to < 70.1; Q2, 70.1 to < 88.3; Q3, 88.3 to < 98.8; Q4, 98.8 to 164 mL/min/1.73 m^2^; at day 3 post-EVT, Q1, 8.4 to < 73.4; Q2, 73.4 to < 91.0; Q3, 91.0 to < 101; Q4, 101 to 149 mL/min/1.73 m^2^. Q1 represents the lowest eGFR values and Q4 the highest. eGFR, estimated glomerular filtration rate; EVT, endovascular therapy; HT, hemorrhagic transformation; sICH, symptomatic intracerebral hemorrhage.

The association between post-EVT eGFR and hemorrhagic complications demonstrated dynamic changes over time. While moderate-to-severe renal impairment on days 1 and 3 was not significantly associated with overall HT incidence (*P* = 0.105; *P* = 0.021), it was significantly associated with increased rates of sICH on both day 1 (*P* = 0.021) and day 3 (*P* < 0.001). Quartile analysis revealed that patients in the lowest eGFR Q1 on day 3 had the highest proportion of sICH (35.82%), although the between-group difference did not reach statistical significance (*P* = 0.060). Similarly, no statistically significant associations were observed for HT events across quartiles (*P* = 0.480).

### 3.4 Multivariable analysis of the association between eGFR and in-hospital mortality

To further evaluate the independent association between eGFR and in-hospital mortality, we constructed three progressively adjusted multivariable logistic regression models (Table 5). Continuous variable analysis demonstrated that baseline eGFR had no significant association with in-hospital mortality risk (Model 3: OR = 0.99, 95% CI: 0.97–1.01, *P* = 0.223). However, post-EVT eGFR levels showed increasingly strong associations with in-hospital mortality. After comprehensive adjustment, each 1 mL/min/1.73 m^2^ increase in eGFR on day 1 post-EVT was associated with a 2% reduction in in-hospital mortality risk (Model 3: OR = 0.98, 95% CI: 0.96–0.99, *P* = 0.002). By day 3 post-EVT, this association strengthened further, with each 1 mL/min/1.73 m^2^ increase in eGFR associated with a 3% reduction in mortality risk (Model 3: OR = 0.97, 95% CI: 0.95–0.98, *P* < 0.001).

**TABLE 5:** Multivariable logistic regression analysis of the association between eGFR at different time points and in-hospital mortality in patients with acute ischemic stroke after endovascular therapy.

Variables	Model 1		Model 2		Model 3	
	OR (95% CI)	*P*-value	OR (95% CI)	*P*-value	OR (95% CI)	*P*-value
eGFR day 0	0.99 (0.98–1.00)	0.095	1.00 (0.98–1.01)	0.610	0.99 (0.97–1.01)	0.223
eGFR day 1	0.98 (0.96–0.99)	<0.001	0.98 (0.96–0.99)	0.001	0.98 (0.96–0.99)	0.002
eGFR day 3	0.97 (0.96–0.98)	<0.001	0.97 (0.96–0.98)	<0.001	0.97 (0.95–0.98)	<0.001
**eGFR day 0**						
≥60	Reference	–	Reference	–	Reference	–
<60	0.88 (0.39–1.98)	0.751	0.63 (0.26–1.49)	0.288	0.72 (0.29–1.77)	0.471
**eGFR day 1**						
≥ 60	Reference	–	Reference	–	Reference	–
<60	2.85 (1.46–5.57)	0.002	2.54 (1.23–5.25)	0.012	2.43 (1.11–5.32)	0.027
**eGFR day 3**						
≥60	Reference	–	Reference	–	Reference	–
<60	5.04 (2.61–9.71)	<0.001	4.51 (2.20–9.27)	<0.001	4.34 (1.98–9.50)	<0.001
**eGFR day 0**						
Q1	Reference	–	Reference	–	Reference	–
Q2	1.08 (0.50–2.37)	0.842	1.15 (0.51–2.61)	0.730	1.24 (0.52–2.93)	0.626
Q3	0.48 (0.19–1.21)	0.121	0.62 (0.23–1.65)	0.340	0.50 (0.18–1.40)	0.186
Q4	0.61 (0.26–1.45)	0.266	0.99 (0.33–2.97)	0.990	0.53 (0.16–1.78)	0.306
P for trend	–	0.107	–	0.690	–	0.191
**eGFR day 1**						
Q1	Reference	–	Reference	–	Reference	–
Q2	0.60 (0.28–1.28)	0.185	0.64 (0.29–1.40)	0.264	0.62 (0.27–1.44)	0.264
Q3	0.27 (0.11–0.68)	0.005	0.33 (0.12–0.88)	0.027	0.29 (0.10–0.83)	0.021
Q4	0.27 (0.11–0.67)	0.005	0.26 (0.08–0.84)	0.024	0.27 (0.08–0.94)	0.039
P for trend	–	<0.001	–	0.008	–	0.012
**eGFR day 3**						
Q1	Reference	–	Reference	–	Reference	–
Q2	0.41 (0.19–0.86)	0.019	0.43 (0.20–0.94)	0.033	0.47 (0.21–1.07)	0.073
Q3	0.17 (0.07–0.44)	<0.001	0.17 (0.06–0.46)	<0.001	0.20 (0.07–0.57)	0.003
Q4	0.11 (0.04–0.33)	<0.001	0.08 (0.02–0.29)	<0.001	0.07 (0.02–0.29)	< 0.001
P for trend	–	<0.001	–	<0.001	–	< 0.001

Data are presented as odds ratios (ORs) with 95% confidence intervals (CIs). For quartile analyses, patients were categorized based on eGFR distribution at each time point: at admission (day 0), Q1, 19.5 to < 68.5; Q2, 68.5 to < 87.5; Q3, 87.5 to < 98.6; Q4, 98.6 to 145 mL/min/1.73 m^2^; at day 1 post-EVT, Q1, 12.0 to < 70.1; Q2, 70.1 to < 88.3; Q3, 88.3 to < 98.8; Q4, 98.8 to 164 mL/min/1.73 m^2^; at day 3 post-EVT, Q1, 8.4 to < 73.4; Q2, 73.4 to < 91.0; Q3, 91.0 to < 101; Q4, 101 to 149 mL/min/1.73 m^2^. Q1 represents the lowest eGFR values (reference group) and Q4 the highest. CI, confidence interval; eGFR, estimated glomerular filtration rate; EVT, endovascular therapy; NIHSS, National Institutes of Health Stroke Scale; OR, odds ratio.

When analyzed as a dichotomized variable with 60 mL/min/1.73 m^2^ as the threshold, baseline moderate-to-severe renal impairment (eGFR < 60) showed no significant difference in mortality risk compared to patients with normal renal function (Model 3: OR = 0.72, 95% CI: 0.29–1.77, *P* = 0.471). However, post-EVT renal impairment demonstrated significantly increased mortality risk. After comprehensive adjustment, patients with eGFR < 60 on day 1 post-EVT had approximately 2.4-fold higher mortality risk than those with normal eGFR (Model 3: OR = 2.43, 95% CI: 1.11–5.32, *P* = 0.027), which further increased to 4.3-fold by day 3 (Model 3: OR = 4.34, 95% CI: 1.98–9.50, *P* < 0.001).

Quartile analysis further revealed a non-linear association pattern and dose-response relationship between eGFR and in-hospital mortality. Baseline eGFR quartiles showed no significant association with mortality risk, with trend analysis also failing to reach statistical significance (P for trend = 0.191). After comprehensive adjustment (Model 3), higher eGFR quartiles on day 1 post-EVT demonstrated significantly reduced mortality risk; compared to Q1, the Q3 group had a 71% lower risk (OR = 0.29, 95% CI: 0.10–0.83, *P* = 0.021) and the Q4 group had a 73% lower risk (OR = 0.27, 95% CI: 0.08–0.94, *P* = 0.039), exhibiting a clear dose-response relationship (P for trend = 0.012).

The most pronounced association was observed in the day 3 post-EVT eGFR quartile analysis, where increasing eGFR quartile levels corresponded to a stepwise decrease in in-hospital mortality risk. After comprehensive adjustment (Model 3), compared to Q1, the Q3 group had an 80% lower risk (OR = 0.20, 95% CI: 0.07–0.57, *P* = 0.003) and the Q4 group had a 93% lower risk (OR = 0.07, 95% CI: 0.02–0.29, *P* < 0.001), with a strong dose-response relationship (P for trend < 0.001).

### 3.5 Non-linear association between eGFR and in-hospital mortality

**FIGURE 2:**
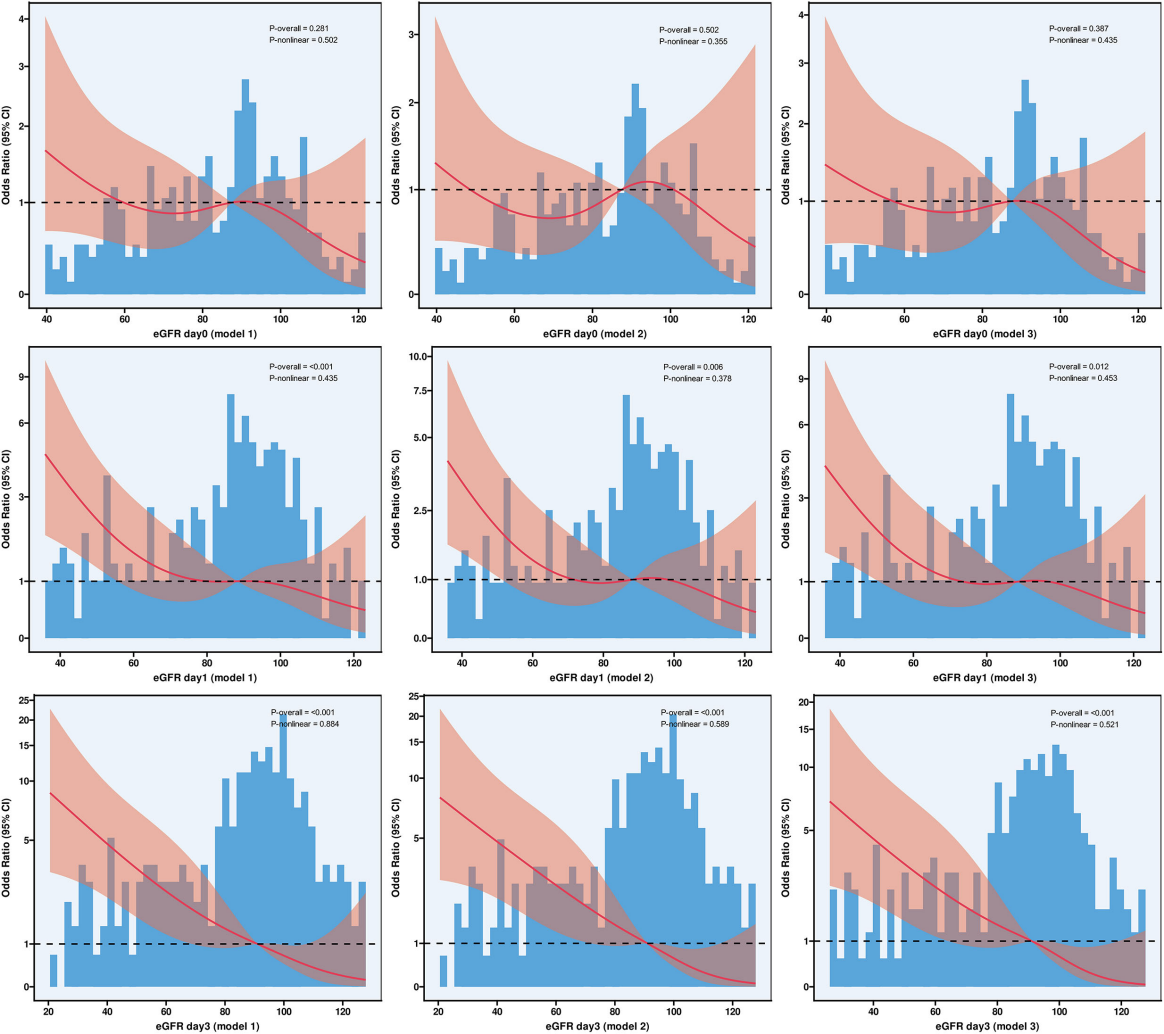
Association between estimated glomerular filtration rate and in-hospital mortality: dose-response analysis using restricted cubic splines. Restricted cubic spline curves illustrate the association between eGFR and in-hospital mortality risk at admission (day 0) and on days 1 and 3 post-EVT across three progressively adjusted models. Model 1, unadjusted; Model 2, adjusted for age, smoking status, atrial fibrillation, and coronary heart disease; Model 3, additionally adjusted for baseline NIHSS score, puncture-to-reperfusion time, and laboratory parameters (lymphocyte count, platelet count, and aspartate aminotransferase). The reference value (odds ratio = 1.0) was set at the cohort median eGFR for each time point. Solid lines represent point estimates of odds ratios; shaded areas represent 95% CIs. Histograms display the distribution of eGFR values. Knots were placed at the 5th, 35th, 65th, and 95th percentiles. P-overall, significance of overall association; P-non-linear, significance of non-linear component. CI, confidence interval; eGFR, estimated glomerular filtration rate; EVT, endovascular therapy; NIHSS, National Institutes of Health Stroke Scale.

To thoroughly investigate potential non-linear relationships between eGFR and in-hospital mortality, we conducted RCS analyses at different time points (Figure 2). These analyses employed three progressively adjusted models. At baseline, the association between eGFR and in-hospital mortality did not demonstrate significant overall non-linear patterns across all three models. The relationship between day 1 eGFR and in-hospital mortality exhibited stronger associations, with all models showing significant overall effects (P-overall values for models 1, 2, and 3 were < 0.001, 0.006, and 0.012, respectively). The RCS revealed a predominantly linear inverse relationship, with mortality risk progressively increasing as eGFR decreased. Tests for non-linearity were not significant (P-non-linear values for models 1, 2, and 3 were 0.435, 0.376, and 0.453, respectively).

The most pronounced association was observed with day 3 eGFR, which demonstrated strong and consistent associations with in-hospital mortality across all adjusted models (all P-overall values < 0.001). Mortality risk significantly increased as eGFR decreased. This relationship was predominantly linear, indicating a consistent dose-response relationship between eGFR decline and increased mortality risk.

### 3.6 Subgroup analysis

**FIGURE 3:**
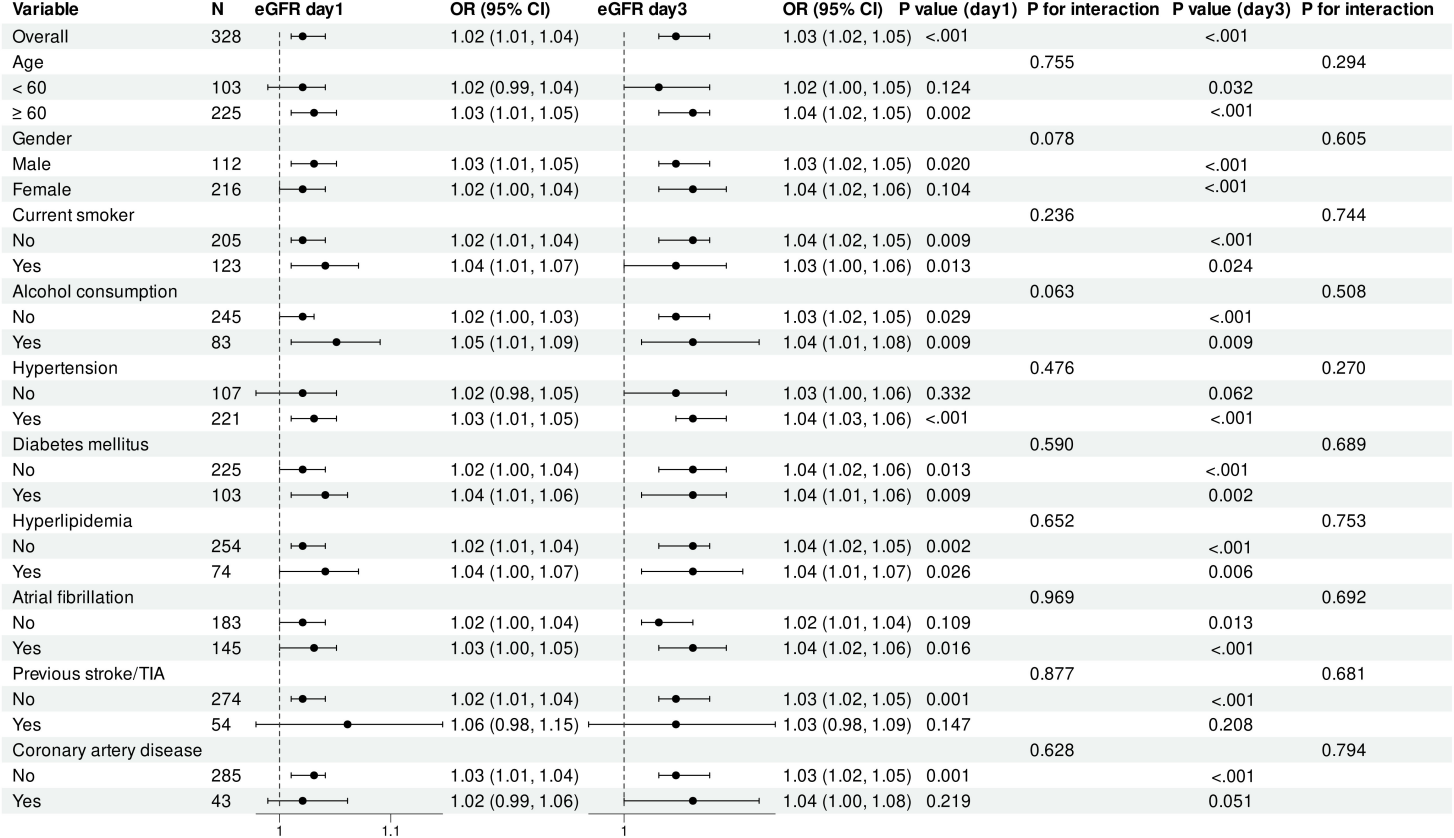
Association between estimated glomerular filtration rate decline and in-hospital mortality across patient subgroups. Forest plot displays odds ratios (ORs) with 95% confidence intervals (CIs) for the association between each 1 mL/min/1.73 m^2^ decrease in eGFR on days 1 and 3 post-EVT and in-hospital mortality across predefined subgroups. ORs were derived from multivariable logistic regression models adjusted for age, smoking status, atrial fibrillation, coronary heart disease, baseline NIHSS score, puncture-to-reperfusion time, lymphocyte count, platelet count, and aspartate aminotransferase. *P*-values indicate the significance of the association within each subgroup; *P*-values for interaction assess the heterogeneity of associations across subgroups. The vertical dashed line represents the null value (OR = 1.0). CI, confidence interval; eGFR, estimated glomerular filtration rate; EVT, endovascular therapy; NIHSS, National Institutes of Health Stroke Scale; TIA, transient ischemic attack.

To assess the consistency of the association between eGFR and in-hospital mortality across different subpopulations, we conducted subgroup analyses based on key demographic characteristics and vascular risk factors (Figure 3). In the total cohort, each 1 mL/min/1.73 m^2^ decrease in eGFR was associated with a 2% increase in in-hospital mortality risk on day 1 (OR = 1.02, 95% CI: 1.01–1.04, *P* < 0.001) and a 3% increase on day 3 (OR = 1.03, 95% CI: 1.02–1.05, *P* < 0.001). Age-stratified analysis revealed that the association between eGFR and mortality was more significant in patients aged ≥ 60 years (day 1: *P* = 0.002; day 3: *P* < 0.001), while in patients < 60 years, only day 3 eGFR showed a significant association (*P* = 0.032). Gender-stratified analysis demonstrated that the association between eGFR and mortality was more consistent in male patients (day 1: *P* = 0.020; day 3: *P* < 0.001), whereas in female patients, this association reached significance only on day 3 (*P* < 0.001). The association between eGFR and mortality was evident across subgroups defined by smoking and alcohol status, diabetes, and hyperlipidemia, although with some differences in effect magnitude. Notably, in patients without hypertension, this association did not reach statistical significance (day 1: *P* = 0.332; day 3: *P* = 0.062).

Interaction tests across all subgroups showed no significant differences (all P for interaction > 0.05), suggesting that the association between eGFR and in-hospital mortality was generally consistent across subgroups. Overall, day 3 eGFR demonstrated stronger and more consistent associations than day 1 eGFR across almost all subgroups.

## 4 Discussion

In this study, we systematically evaluated the association between dynamic eGFR changes and in-hospital mortality as well as hemorrhagic complications in patients with anterior circulation LVO-AIS undergoing EVT. Our principal findings include the following: First, dynamic changes in eGFR, rather than baseline levels at admission, were independently associated with in-hospital mortality after EVT. This association demonstrated clear time-dependency and a dose-response relationship, reaching peak strength on day 3 post-EVT. Second, patients who died during hospitalization exhibited a pattern of sustained eGFR decline, whereas survivors showed a modest upward trend. Additionally, moderate-to-severe renal dysfunction post-EVT was significantly associated with increased risk of sICH, but not with HT in general. Collectively, these findings underscore that dynamic eGFR monitoring may provide superior prognostic information compared with single time-point assessment.

The existing literature on the association between eGFR and clinical outcomes in AIS remains controversial. From a risk perspective, low eGFR levels constitute an independent risk factor for cerebrovascular events in healthy populations. Oh et al. (2021), in their analysis based on the Korean National Sample Cohort, demonstrated that individuals with eGFR < 45 had significantly higher incidence of ischemic stroke compared to those with normal renal function. Regarding acute phase prognosis, multiple studies have confirmed that lower eGFR levels correlate with various adverse clinical outcomes in AIS (Liu et al., 2020). Kim et al. (2015) reported in their cohort study that baseline eGFR < 30 was significantly associated with early neurological deterioration during hospitalization (OR = 2.37) and poor functional outcomes at 3 months (OR = 2.98). Similarly, Lee et al. (2013) observed a significant association between severe renal impairment and increased risk of HT in AIS patients (OR = 2.90). Recently, some researchers have begun to focus on the prognostic significance of dynamic eGFR changes. Shi et al. (2019) found that both decreased baseline eGFR (HR = 4.17) and eGFR decline at 24 h post-thrombolysis (HR = 5.88) were significantly associated with increased 3-month all-cause mortality in AIS patients receiving intravenous thrombolysis. However, a cohort study including 1,420 patients reached different conclusions (Eun et al., 2021). This study found in univariate analysis that reduced eGFR was significantly associated with poor functional outcomes at discharge and risk of in-hospital mortality, but showed no clear association with neurological deterioration or HT; in multivariate analysis adjusting for potential confounding factors, eGFR reduction was not independently associated with adverse clinical outcomes.

Unlike previous studies primarily focusing on general AIS patients or those receiving intravenous thrombolysis, we concentrated on the specific high-risk population of anterior circulation LVO patients undergoing EVT. These patients face unique and complex pathophysiological challenges, including more extensive initial ischemic injury, broader ischemic penumbra, and higher risk of reperfusion-related complications. Consequently, the pattern of association between renal function and outcomes may differ from that in conventional stroke populations. Nevertheless, research on the relationship between eGFR and outcomes in the context of EVT remains quite limited. Yao et al. (2021) reported that pre-procedural eGFR reduction was independently associated with poor 3-month clinical outcomes in AIS patients undergoing EVT (OR = 1.059). However, that study only assessed eGFR at a single pre-procedural time point. Our approach of monitoring eGFR at three consecutive time points provided a more comprehensive capture of the dynamic interaction between renal function and brain injury during the acute phase of stroke.

Multiple potential pathophysiological mechanisms may explain the association between eGFR and adverse outcomes in EVT patients. First, patients with chronic renal dysfunction frequently present with a higher prevalence of traditional cerebrovascular risk factors, including hypertension, dyslipidemia, and diabetes mellitus, which collectively create a pathological milieu conducive to poor prognosis (Bobot et al., 2023). Second, the imbalance between coagulation and anticoagulation systems in renal dysfunction states may increase the risk of both thrombus formation and hemorrhage, with the latter particularly exacerbating intracerebral hemorrhagic complications in patients with ischemic stroke (Kaw and Malhotra, 2006). More importantly, non-traditional risk factors such as the accumulation of uremic toxins may significantly influence ischemic stroke outcomes (Liabeuf et al., 2021). Existing research has confirmed that BBB dysfunction substantially enhances intracerebral inflammatory responses, particularly in the late phase after stroke. CKD patients commonly exhibit a state of low-grade systemic inflammation, characterized by persistently elevated levels of pro-inflammatory cytokines (IL-1β, IL-6, and TNF-α), which may further amplify the intensity and duration of post-stroke neuroinflammatory responses, aggravating brain tissue damage (Afsar et al., 2016).

The relationship between AKI and stroke outcomes also warrants attention. During stroke hospitalization, the incidence of AKI is approximately 12%, and multiple studies have confirmed its strong association with increased post-stroke mortality and poor neurological outcomes (Zorrilla-Vaca et al., 2018). Animal experimental studies indicate that AKI can induce systemic inflammatory cascade reactions, promoting the release of various pro-inflammatory cytokines (IL-6, IL-1β) and free radical production (Hoke et al., 2007). Additionally, AKI enhances neuroinflammatory responses associated with astrocyte proliferation in the brain while reducing BBB integrity through disruption of endothelial tight junction protein expression. These pathological alterations act synergistically to potentially exacerbate secondary brain tissue damage following ischemic stroke (Caillard et al., 2022; Liu et al., 2008). In our cohort, patients who died during hospitalization generally exhibited a significant downward trend in eGFR, potentially reflecting the occurrence of AKI and its detrimental impact on neurological outcomes. However, we did not observe a statistically significant association between baseline eGFR levels and in-hospital mortality risk, which may be partially attributed to methodological limitations. Creatinine-based eGFR calculations in emergency settings are influenced by multiple factors, including patients’ recent dietary protein intake, fluctuations in hydration status, and metabolic alterations during acute stress, which may affect the accuracy of baseline eGFR assessment.

Our findings have significant clinical applications. First, our results strongly support implementing a dynamic renal function monitoring strategy following EVT, rather than relying solely on baseline assessment at admission. In particular, eGFR measurement on day 3 post-EVT may provide the most valuable prognostic information. Second, patients exhibiting progressive decline in eGFR should be identified as a high-risk population requiring enhanced monitoring and consideration for early intervention. These patients may benefit from more frequent neurological assessments, more aggressive fluid management, and early preventive interventions. Third, our study provides a foundation for future integration of renal function parameters into prognostic assessment models for EVT patients. Traditional stroke prognostic tools such as NIHSS and ASPECTS scores primarily focus on neurological and imaging parameters, whereas EVT patients face more complex systemic challenges (Gao et al., 2025). Incorporating dynamic eGFR changes into existing predictive models could significantly enhance their ability to identify high-risk patients, thereby enabling more precise risk stratification. Moreover, although our observational study indicates a significant association between eGFR decline and adverse outcomes, it remains unclear whether active nephroprotective strategies could improve patient prognosis. Rigorously designed randomized controlled trials are needed to evaluate the impact of perioperative renal protection protocols on neurological outcomes in EVT patients.

Several limitations of this study warrant consideration. First, as a single-center retrospective cohort study, inherent selection and information biases are unavoidable. Despite our comprehensive multivariate adjustments, unmeasured confounding factors may still exist. Our findings require validation in larger, multicenter cohorts of EVT patients. Second, creatinine-based eGFR calculations have intrinsic limitations, particularly in patients during the acute phase. Creatinine, as an indirect marker, is influenced by multiple factors including age, sex, muscle mass, nutritional status, and medications. Furthermore, our study cannot establish a definitive causal relationship between eGFR changes and clinical outcomes. It remains unclear whether eGFR decline may have specific effects on stroke outcomes per se, or whether it is related to the overall severity of stroke patients, as it is known to be an independent risk factor for mortality in hospitalized and intensive care patients, as well as a risk factor for confusion and coma in critically ill patients.

## 5 Conclusion

To our knowledge, this is the first study to demonstrate that dynamic eGFR decline is independently associated with in-hospital mortality in patients with anterior circulation LVO-AIS undergoing EVT. Patients who died exhibited a pattern of progressive eGFR decline, whereas survivors demonstrated a modest upward trajectory. These findings suggest that dynamic eGFR monitoring in EVT patients may provide more valuable prognostic information than single time-point assessment. Future multicenter studies are warranted to validate these findings and explore whether targeted interventions to preserve renal function could improve clinical outcomes in this high-risk population.

## Data Availability

The datasets presented in this article are not readily available because the datasets used and/or analyzed during the current study are available from the corresponding author upon reasonable request. Requests to access the datasets should be directed to Renjing Zhu.
