# Supplementary material for: Dynamic decline in estimated glomerular filtration rate associated with in-hospital mortality risk in acute ischemic stroke patients after endovascular therapy: evidence from a Chinese stroke center
Source: Front Aging Neurosci. 2025 Nov 6;17:1598371. doi: 10.3389/fnagi.2025.1598371 (PMC12631443; doi:10.3389/fnagi.2025.1598371)
Supplement: Supplementary file 1 [file Table_1.docx]

| **Supplementary Table 1.** Multicollinearity assessment of variables included in the multivariable logistic regression models | | |
| --- | --- | --- |
| **Variables** | Tolerance | VIF |
| eGFR day0 | 0.250 | 3.997 |
| eGFR day1 | 0.165 | 6.058 |
| eGFR day3 | 0.245 | 4.075 |
| Age | 0.574 | 1.740 |
| Current smoker | 0.912 | 1.096 |
| Atrial fibrillation | 0.687 | 1.456 |
| Coronary artery disease | 0.916 | 1.092 |
| Baseline NIHSS score | 0.876 | 1.141 |
| Puncture to recanalization time | 0.839 | 1.192 |
| Lymphocyte | 0.843 | 1.186 |
| Platelet count | 0.882 | 1.134 |
| Aspartate aminotransferase | 0.973 | 1.028 |
| Multicollinearity diagnostics for variables included in the adjusted models predicting in-hospital mortality after endovascular therapy for anterior circulation large vessel occlusion acute ischaemic stroke. Variance inflation factor (VIF) values <10 and tolerance values >0·1 indicate acceptable multicollinearity levels. NIHSS=National Institutes of Health Stroke Scale. | | |
